# The Role of Edible Bulbous Layers on Macro, Micro, and Heavy Metal Contents of Leek (*Allium porrum*) Plant

**DOI:** 10.1007/s12011-024-04181-w

**Published:** 2024-04-17

**Authors:** Fahad AlJuhaimi, Duygu Akçay Kulluk, Isam A Mohamed Ahmed, Fatma Gökmen Yılmaz, Emad Karrar, Mehmet Musa Özcan

**Affiliations:** 1 Department of Food Science & Nutrition, College of Food and Agricultural Sciences, King Saud University, Riyadh, Saudi Arabia; 2 Department of Soil Science and Plant Nutrition, Faculty of Agriculture, Selcuk University, Konya, 42031 Turkey; 3 College of Ocean Food and Biological Engineering, Jimei University, Xiamen, 361021 China; 4 Faculty of Agriculture, Department of Food Engineering, Selcuk University, Konya, 42031 Turkey

**Keywords:** Leek, Different parts, Layers, Element contents, Heavy metals, ICP-OES

## Abstract

In this study, the degree of accumulation of biogenic element and heavy metal contents of different parts and edible layers of leeks cultivated in Konya in Turkey was revealed. The amounts of P and K of leek were determined from 154.69 (leaf top of leek) and 985.05 mg/kg (root of leek) to 1377.63 (onion part of leek) and 2688.50 mg/kg (root of leek), respectively. P and K contents of leek layers changed from 139.45 (1st layer) and 446.63 mg/kg (7th layer) to 1596.69 (2nd layer) and 2201.53 mg/kg (4th layer), respectively. While Ca amounts of leek parts vary between 577.09 (leaf of leek) and 666.87 mg/kg (root of leek), Mg contents of leek parts were determined between 130.70 (onion part of leek) and 264.58 mg/kg (root of leek). All of the macro elements were detected in the highest amount in the root of the leek, followed by the leaf and bulb parts in decreasing order. Fe and Zn contents of different parts of leeks varied from 0.506 (onion part of leek) and 22.71 mg/kg (root of leek) to 1.53 (leaf top of leek) and 5.85 mg/kg (root of leek), respectively. In general, the heavy metals found in the highest amount both in different parts of the leek and in the edible bulbous layers were As and Ba. The layers of the leeks are rich in potassium, phosphorus, iron, and zinc.

**Supplementary Information:**

The online version contains supplementary material available at 10.1007/s12011-024-04181-w.

## Introduction

 Leek (*Allium porrum* L.) is one of the most commercially produced vegetables in the world [1–3]. Leek is an edible biennial herb belonging to the Alliaceae family, originating from Asia and grown for consumption in ancient Egypt, Greece, and Rome. Leek is consumed as an appetizer or as a thickener in soups, as well as cooked with olive oil. Besides, people have also reported using leeks for insect bites and even bleeding [4]. Fruits and vegetables, which have an important role in human nutrition, contain many components that include health benefits and disease-preventing factors [5]. Fresh leeks contain many organosulfur compounds, nitrates, flavonoids, polysaccharides, and glucosinolates with flavor characteristics [6–8]. As a result of epidemiological and laboratory studies, allium vegetables have an anti-tumor effect, and leek consumption has a reducing effect on the risk of prostate [9, 10]. The fact that organic substances and other minerals in plants can affect the amount of trace elements to be absorbed from the intestines increases the importance of plant composition [11]. Vegetables, rich in many antioxidants and nutrients, are essential for human health and contribute significantly to the total daily requirement of potassium, magnesium, and phosphorus in the human diet [12]. The leaves and long white blanched stem of the leek can be eaten cooked as well as consumed as salads [13]. Leek due to its benefits to human health accounts for approximately 13.5% of the total agricultural production in Turkey [14]. Leek, which has the ability to accumulate significant amounts of potassium and iron, is also an important source of minerals such as zinc, calcium, phosphorus, copper, sodium, manganese, and magnesium [15–17]. Additionally, leeks are rich in various biologically active compounds such as glucosinolates and pectic polysaccharides and a metabolite, S-alkenyl-L-cysteine sulfoxides [16]. In this study, the main target was to investigate the amount of heavy metals as well as biogenic elements accumulated in the parts of leeks. The aim of this study is to determine the degree of accumulation of biogenic element and heavy metal contents of different parts and edible layers of leeks cultivated in Konya in Turkey.

## Materials and Methods

### Material

**Fig. 1:**
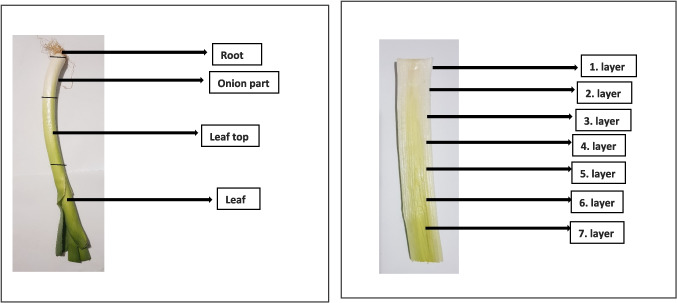
Different parts and layers of leeks used in this study

Leeks (*Allium porrum* L.) were provided from a local market in Konya in 2023. Before the analysis, the leek plant parts and layers (Fig. 1) were washed with distilled water, and then the different parts and layers and roots were cut. Each part and layer were thoroughly crushed in the blender and homogenized. HNO_3_ and H_2_O_2_ are analytical grade and Merck company (Darmstadt, Germany).

### Method

#### Determination of Moisture

The moisture results of the parts of leek were detected at 70 °C/48 h using an oven till a constant weight  [18].

#### Macro, Micro, and Heavy Metal Contents of Leek Samples

After 0.2 g leek sample was incinerated in a microwave device (Cem MARSXpress 6 One Touch Model, USA) at 210 °C and 200 PSI pressure in 5 ml of concentrated HNO_3_ and 2 ml of H_2_O_2_ (30% w/v), the volumes of the dissolved samples were completed to 20 ml with deionized water. Then, heavy metal concentrations in the samples were analyzed with ICP-OES  [19, 20].

#### Statistical Analysis

The JMP (JMP, SAS Institute, Cary, NC) statistical program was used for the statistical analysis of results obtained. Statistically significant differences were determined by the analysis of variance (ANOVA) procedure in all data (*p* < 0.01)  [21].

## Results and Discussion

The moisture and biogenic element results of several parts and layers of leek plants are shown in Table 1. Results obtained exhibited some changes based on leek parts. The moisture of leek parts were measured between 84.98 (root of leek) and 92.11% (leaf top of leek). As seen in Table 1, the highest dry matter was found in the root part of the leek. The moisture of the layers belonging to the most consumable parts of the leek were determined between 86.52 (3rd layer) and 91.84% (4th layer). Partial differences (*p* < 0.01) were observed between the mineral contents of the leek layers. P and K values of leek parts were determined from 154.69 (leaf top of leek) and 985.05 mg/kg (root of leek) to 1377.63 (onion part of leek) and 2688.50 mg/kg (root of leek), respectively. Also, P and K amounts of leek layers changed from 139.45 (1st layer) and 446.63 mg/kg (7th layer) to 1596.69 mg/kg (2nd layer) and 2201.53 mg/kg (4th layer), respectively. While Ca quantities of leek parts vary between 577.09 (leaf of leek) and 666.87 mg/kg (root of leek), Mg results of leek parts were assessed to be between 130.70 (onion part of leek) and 264.58 mg/kg (root of leek). In addition, Ca and Mg values of leek layers were measured from 242.70 (4th layer) and 454.75 mg/kg (7th layer) to 84.69 (2nd layer) and 169.08 mg/kg (5th layer), respectively. S amounts of leek parts and layers were determined from 335.32 (leaf top of leek) and 954.04 mg/kg (root of leek) to 287.13 (2nd layer) and 617.75 mg/kg (6th layer), respectively. As seen in Table 1, all of the macro elements were detected in the highest amount in the root of the leek, followed by the leaf and bulbous parts in decreasing order. In the leek layers, the highest P and K amounts were determined in the 4th layer of the leek. In general, it was observed that the amounts of macro elements were accumulated in different amounts in different layers of leek. The greater presence of macro elements in leek root is probably due to retention of soil soluble elements in root cells. The element difference in the leek layers may depend on the amount of elements that each layer can take from the stem cells through the transmission channels from the root of the leek. In general, the amount of macro elements of different parts of the leek was slightly higher than that of the leek layers, although there were some differences.

**Table 1:** Moisture (%) and macro element (mg/kg) contents of different parts and layers of leeks

Sample	Moisture	P	K	Ca	Mg	S
Root of leek	84.98 ± 3.87 **b**	985.05 ± 123.60 **a**	2688.50 ± 263.75 **a**	666.87 ± 101.04	264.58 ± 34.51 **a**	954.04 ± 69.83 **a**
Onion part of leek	90.23 ± 0.759 **a**	245.72 ± 69.49 **b**	1377.63 ± 243.36 **c**	614.22 ± 87.08	130.70 ± 9.27 **b**	429.72 ± 10.20 **b**
Leaf top of leek	92.11 ± 0.032 **a**	154.69 ± 20.46 **b**	1421.73 ± 190.60 **c**	628.72 ± 22.12	158.10 ± 22.72 **b**	335.32 ± 5.08 **c**
Leaf of leek	92.08 ± 0.130 **a**	195.24 ± 31.21 **b**	2250.97 ± 159.89 **b**	577.09 ± 23.62	158.16 ± 12.25 **b**	377.07 ± 49.92 **bc**
Sample	Moisture	P	K	Ca	Mg	S
1. Layer of leek	89.65 ± 1.20 **c**	139.45 ± 24.87 **d**	1867.15 ± 81.34 **c**	429.16 ± 71.94 **a**	119.57 ± 20.29 **bc**	438.43 ± 44.50 **b**
2. Layer of leek	90.08 ± 1.87 **bc**	174.90 ± 29.57 **cd**	1596.69 ± 42.32 **d**	244.32 ± 21.08 **c**	84.69 ± 10.43 **d**	287.13 ± 31.75 **c**
3. Layer of leek	86.52 ± 0.231 **d**	178.56 ± 6.07 **cd**	1838.37 ± 105.71 **c**	287.82 ± 15.53 **bc**	96.77 ± 9.16 **cd**	290.35 ± 11.71 **c**
4. Layer of leek	91.84 ± 0.297 **a**	250.53 ± 21.64 **bc**	2201.53 ± 84.13 **a**	242.70 ± 10.32 **c**	121.57 ± 5.20 **b**	378.87 ± 11.29 **b**
5. Layer of leek	90.91 ± 0.381 **abc**	315.73 ± 15.41 **b**	2155.18 ± 105.04 **ab**	295.08 ± 63.44 **bc**	169.08 ± 16.38 **a**	402.34 ± 26.04 **b**
6. Layer of leek	91.30 ± 0.421 **ab**	444.46 ± 71.22 **a**	2006.27 ± 173.09 **abc**	393.51 ± 64.65 **ab**	159.74 ± 11.35 **a**	617.75 ± 45.66 **a**
7. Layer of leek	91.01 ± 0.415 **abc**	446.63 ± 119.53 **a**	1972.82 ± 212.19 **bc**	454.75 ± 123.06 **a**	99.28 ± 17.92 **bcd**	381.18 ± 72.98 **b**

*p* < 0.01

The micro elements of the parts and layers of leek plants are presented in Table 2. Fe and Zn contents of different parts of leeks varied from 0.506 (onion part of leek) and 22.71 mg/kg (root of leek) to 1.53 (leaf top of leek) and 5.85 mg/kg (root of leek), respectively. Fe and Zn values of different layers of leek plants were measured from 0.903 (1st layer) and 8.27 mg/kg (7th layer) to 0.234 (7th layer) and 3.02 mg/kg (2nd layer), respectively. The Zn contents of the 1st and 2nd layers of the leek were found to be very close to each other. Fe amounts of three, four, and five layers of leeks were found to be similar to each other. While Cu contents of leek parts change between 0.700 (leaf top of leek) and 2.99 mg/kg (root of leek), Mn contents of leek parts were recorded between 0.823 (onion part of leek) and 11.99 mg/kg (root of leek). Fe and Mn contents of “onion part of leek” and “leaf top of leek” parts of leek were found to be very close to each other. Cu and Mn contents of leek layers were reported from 0.143 (7th layer) and 0.525 mg/kg (5th later) to 0.104 (7th layer) and 0.967 mg/kg (5th layer), respectively. The highest Zn, Cu, and Mn amounts were determined in the 5th layer of leek. The lowest Cu and Mn were detected in the 7th layer of leek. The highest Zn was determined in the outer 1st, 2nd, and 3rd layers of the leek. In general, the micro elements of the leek parts (except Fe) were found to be slightly higher than the micro elements of the leek layers (layers). The micro elements of the leek parts were determined in the highest “root of leek” part.

**Table 2:** Micro element contents of different parts and layers of leeks (mg/kg)

Sample	Fe	Zn	Cu	Mn
Root of leek	22.71 ± 3.25 **a**	5.85 ± 0.406 **a**	2.99 ± 0.507 **a**	11.99 ± 1.59 **a**
Onion part of leek	0.506 ± 0.038 **b**	2.16 ± 0.180 **b**	0.907 ± 0.078 **b**	0.823 ± 0.041 **b**
Leaf top of leek	0.585 ± 0.084 **b**	1.53 ± 0.095 **c**	0.700 ± 0.022 **b**	0.829 ± 0.126 **b**
Leaf of leek	0.592 ± 0.046 **b**	1.78 ± 0.141 **bc**	0.804 ± 0.138 **b**	1.30 ± 0.223 **b**
Sample	Fe	Zn	Cu	Mn
1. Layer of leek	0.903 ± 0.065 **c**	3.01 ± 0.366 **a**	0.418 ± 0.108 **ab**	0.527 ± 0.080 **b**
2. Layer of leek	2.54 ± 0.159 **b**	3.02 ± 0.176 **a**	0.373 ± 0.096 **b**	0.601 ± 0.050 **b**
3. Layer of leek	3.53 ± 1.07 **b**	2.59 ± 0.336 **b**	0.478 ± 0.133 **ab**	0.610 ± 0.035 **b**
4. Layer of leek	3.29 ± 0.421 **b**	1.96 ± 0.097 **c**	0.479 ± 0.008 **ab**	0.608 ± 0.057 **b**
5. Layer of leek	3.37 ± 0.239 **b**	2.70 ± 0.171 **ab**	0.525 ± 0.021 **a**	0.967 ± 0.083 **a**
6. Layer of leek	8.25 ± 0.846 **a**	0.269 ± 0.025 **d**	0.157 ± 0.020 **c**	0.118 ± 0.018 **c**
7. Layer of leek	8.27 ± 1.71 **a**	0.234 ± 0.050 **d**	0.143 ± 0.011 **c**	0.104 ± 0.021 **c**

*p* < 0.01

**Fig. 2:**
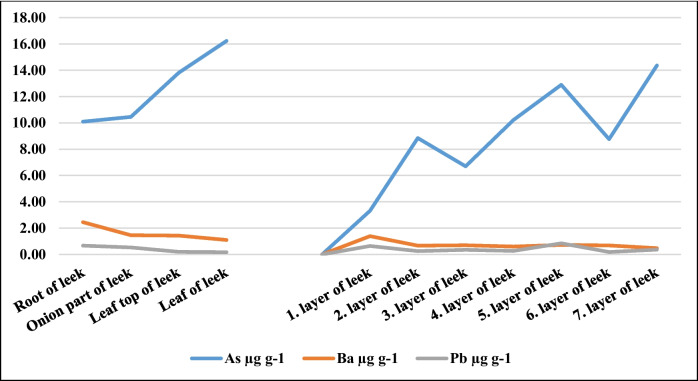
Heavy metals in the highest amount in leek parts and layers

The heavy metal contents and their quantitative values of the parts and layers of leek plants are illustrated in Table 3. As quantities of different parts and layers of leek plants were established from10.10 (root of leek) and 16.23 µg/g (leaf of leek) to 3.31 (1st layer) and 14.36 µg/g (7th layer), respectively. While Ba amounts of different parts of leeks are measured between 1.10 (leaf of leek) and 2.45 µg/g (root of leek), Ba quantities of different layers of leeks were established between 0.478 (7th layer) and 1.39 µg/g (1st layer). Ni and Pb quantities of different parts of leeks were detected from 0.362 (leaf top of leek) and 0.469 µg/g (onion part of leek) to 0.167 (leaf of leek) and 0.667 µg/g (root of leek), respectively. Also, while Ni amounts of different layers of leeks are recorded between 0.232 (5th layer) and 0.407 µg/g (6th layer), Pb contents of leek layers were monitored between 0.187 (6th layer) and 0.844 µg/g (5th layer). Se values of the parts and layers of leek plants were recorded from 0.136 (leaf top of leek) and 0.726 µg/g (root of leek) to 0.247 (2nd layer) and 0.617 µg/g (3rd layer), respectively. Among the different parts of the leek, the highest Cr and Mo were determined in the “leaf of leek” part. Cd amounts of different parts and layers of leeks were reported from 0.013 (leaf of leek) and 0.113 µg/g (onion part of leek) to 0.060 (7th layer) and 0.12 µg/g (2nd layer), respectively. In general, the heavy metals found in the highest amount both in different parts of the leek and in the edible bulbous layers were As and Ba (Fig. 2). Ba content was found at the highest level in the outermost layer (1st layer) and “root of leek” part of leek. Among the leek parts, the lowest Ba, Cd, Co, and Pb were determined in the “leaf of leek” part. Interestingly, As was found in high amounts in each regenerative layer of leek, but the amounts of this element differed between leek layers. The fact that all heavy metals (except As and Ba) are found in very low amounts in different parts and layers of leek makes it advantageous to consume leeks safely. Golubkina et al. [16] reported that pseudo-stem part of leek contained Ca ( 2.81–11.32), K (4.71–51.75), Mg (0.56–2.02), Na (0.12–0.81), P (1.97–3.85 g/kg). Golubkina et al. [16] determined 8.61–21.25 mg/kg B, 0.034–0.290 mg/kg Co, 3.46–7.18 mg/kg Cu, 77–235 mg/kg Fe, 6.39–23.15 mg/kg Mn, and 11.96–27.27 mg/kg Zn in pseudo-stem part of leeks. Al, As, Cd, Cr, Ni, and Pb contents of pseudo-stem part of leeks changed between 8.0 and 137.0 mg/kg, 0.009–0.066 mg/kg, 0.073–0.196 mg/kg, 0.085–0.524 mg/kg, 0.480–1.140 mg/kg, and 0.096–0.894 mg/kg [16]. Latif and El-Aal [22] determined 0.49–0.91% Na, 2.3–5.8% K, 0.5–1.2% Ca, 0.22–0.40% Mg, 0.32–0.45% P, 310–650 ppm Fe, 90–130 ppm Mn, 16–64 ppm Zn, and 9–14 ppm Cu (dw) in leeks. Tepecik et al. [23] reported that plant nutrient contents in the 1st leaf leek were Ca (0.42–0.58%), P (0.38–0.60%), K (4.72–5.79%), Mg (0.40–0.53%), Fe (38.85–51.39 mg/kg), Cu (2.50–5.04 mg/kg), Zn (27.07–39.89 mg/kg), and Mn (16.80-23.54 mg/kg). Otunola et al. [24] determined 54.00 K, 4.10 Mg, 4.10 Na, 26.30 Ca, 5.29 Fe, 10.19P, 0.34 Zn, 0.001 Cu, and 0.001 mg/100 g Mn in garlic. In other study, Ujowundu et al. [25] determined 0.373 Cu, 3.48 Fe), 1.904 Ca, 0.02 Se, and 4.334 mg/100 g Mg in garlic. Koca and Taşcı [26] determined between 7.98 and 14.77% dry matter in leeks. In other study, leeks contained 16,533.87–32,862.46 ppm K, 2491.08–5056.74 ppm Ca, 1176.21–2199.20 ppm P, 631.04–3775.57 ppm Na, 671.79–1882.10 ppm Mg, 21.35–138.81 ppm Fe, 17.65–128.60 ppm Zn, 4.45–25.62 ppm Cu, and 1.54–3.36 ppm Se (dw) in leeks [26]. Generally, the mineral levels of the present study were found compared to results of studies conducted by Latif and El-Aal [22] and Koca and Taşcı [26], but high than those found by Golubkina et al. [16]. In our study, when the element contents of different parts and layers of leek grown under the same geochemical conditions were compared with varieties belonging to related species such as garlic, it was observed that most of the elements were in higher concentrations. Interestingly, significant differences in the content of most elements were observed. It is worth noting that the average levels of heavy metals (except As and Ba) in different parts and layers of leeks in the Konya region are not different from each other. Ca, K, and Mg ensure the development of plants, protect the plant against cold weather conditions, regulate water balance, increase the plant’s resistance to drought, and play an active role in photosynthesis  [27–29]. Additionally, when the presence of biogenic elements and heavy metals in the parts of leek was compared with literature values, significant differences were determined. One of these differences is the relatively high heavy metal content of leeks. The increase in the amount of heavy metals in leeks may have been caused by factors such as fertilization, dirty water and soil, and the distance of the leek growing areas to the industrial zone and air pollution.

**Table 3:** Heavy metal contents of different parts and layers of leeks (µg/g)

Sample	As µg g^−1^	Ba	Cd	Co	Cr	Mo	Ni	Pb	Se
Root of leek	10.10 ± 2.01 **b**	2.45 ± 0.587 **a**	0.089 ± 0.004 **b**	0.304 ± 0.037	0.193 ± 0.021 **b**	0.163 ± 0.018 **a**	0.422 ± 0.042 **ab**	0.667 ± 0.054 **a**	0.726 ± 0.052 **a**
Onion part of leek	10.46 ± 1.84 **b**	1.46 ± 0.148 **b**	0.113 ± 0.011 **a**	0.380 ± 0.091	0.123 ± 0.017 **c**	0.125 ± 0.004 **b**	0.469 ± 0.075 **a**	0.531 ± 0.057 **b**	0.149 ± 0.020 **c**
Leaf top of leek	13.81 ± 0.673 **ab**	1.43 ± 0.226 **b**	0.056 ± 0.014 **c**	0.390 ± 0.063	0.107 ± 0.007 **c**	0.109 ± 0.004 **b**	0.362 ± 0.022 **b**	0.193 ± 0.006 **c**	0.136 ± 0.001 **c**
Leaf of leek	16.23 ± 2.86 **a**	1.10 ± 0.044 **b**	0.013 ± 0.001 **d**	0.298 ± 0.017	0.243 ± 0.037 **a**	0.170 ± 0.010 **a**	0.380 ± 0.033 **ab**	0.167 ± 0.017 **c**	0.481 ± 0.043 **b**
Sample	As	Ba	Cd	Co	Cr	Mo	Ni	Pb	Se
1. Layer of leek	3.31 ± 0.841 **e**	1.39 ± 0.228 **a**	0.091 ± 0.013 **d**	0.381 ± 0.072 **a**	0.195 ± 0.026 **a**	0.091 ± 0.016 **b**	0.367 ± 0.108 **ab**	0.646 ± 0.044 **b**	0.258 ± 0.042 **d**
2. Layer of leek	8.84 ± 1.56 **cd**	0.674 ± 0.191 **bc**	0.129 ± 0.009 **b**	0.218 ± 0.033 **b**	0.219 ± 0.032 **a**	0.107 ± 0.044 **d**	0.283 ± 0.039 **bc**	0.249 ± 0.015 **d**	0.247 ± 0.015 **d**
3. Layer of leek	6.70 ± 1.19 **d**	0.698 ± 0.083 **bc**	0.089 ± 0.005 **de**	0.277 ± 0.034 **b**	0.124 ± 0.015 **bc**	0.127 ± 0.012 **a**	0.322 ± 0.082 **abc**	0.351 ± 0.095 **c**	0.617 ± 0.018 **a**
4. Layer of leek	10.22 ± 1.33 **bc**	0.600 ± 0.052 **bc**	0.075 ± 0.002 **ef**	0.260 ± 0.060 **b**	0.122 ± 0.025 **bc**	0.114 ± 0.009 **a**	0.247 ± 0.026 **c**	0.266 ± 0.023 **d**	0.305 ± 0.027 **c**
5. Layer of leek	12.89 ± 2.49 **ab**	0.725 ± 0.106 **b**	0.167 ± 0.016 **a**	0.276 ± 0.049 **b**	0.158 ± 0.009 **b**	0.074 ± 0.007 **b**	0.232 ± 0.014 **c**	0.844 ± 0.048 **a**	0.258 ± 0.009 **d**
6. Layer of leek	8.76 ± 0.652 **cd**	0.683 ± 0.097 **bc**	0.110 ± 0.005 **c**	0.412 ± 0.015 **a**	0.107 ± 0.016 **c**	0.056 ± 0.009 **c**	0.407 ± 0.080 **a**	0.187 ± 0.018 **d**	0.518 ± 0.006 **b**
7. Layer of leek	14.36 ± 2.72 **a**	0.478 ± 0.057 **c**	0.060 ± 0.005 **f**	0.432 ± 0.048 **a**	0.158 ± 0.002 **b**	0.043 ± 0.008 **c**	0.359 ± 0.016 **ab**	0.356 ± 0.033 **c**	0.505 ± 0.029 **b**

*p* < 0.01

## Conclusion

All of the biogenic elements were detected in the highest amount in the root of the leek, followed by the leaf and bulb parts in decreasing order. The highest Zn, Cu, and Mn amounts were determined in the 5th layer of leek. The lowest Cu and Mn were detected in the 7th layer of leek. The highest Zn was determined in the outer 1st, 2nd, and 3rd layers of the leek. In general, the micro element contents of the leek parts (except Fe) were found to be slightly higher than the micro element contents of the leek layers. Among the leek parts, the lowest Ba, Cd, Co, and Pb were determined in the “leaf of leek” part. It is thought that the amount of fertilizer and pesticide used in the fields should be carefully monitored in order to control heavy metal pollution in vegetables and prevent its spread. According to the results, since the toxic heavy metal contents in leeks are below the maximum allowed limits, there is no harm in consuming these layers. This is an indication that the leeks used in the experiment were grown in a location free of environmental waste. Although there are partial fluctuations, the amounts of these elements are within the limits of international standards since the heavy metals detected in leeks are found in low concentrations. It was thought that leek is an important source of nutrients in terms of macro and micro elements, and leek consumption can contribute to a balanced diet.

## Electronic Supplementary Material

Below is the link to the electronic supplementary material.

Supplementary Material 1

## Data Availability

No datasets were generated or analyzed during the current study.
