# Supplementary material for: The Role of Edible Bulbous Layers on Macro, Micro, and Heavy Metal Contents of Leek (Allium porrum) Plant
Source: Biol Trace Elem Res. 2024 Apr 17;203(1):549–55. doi: 10.1007/s12011-024-04181-w (PMC11700913; doi:10.1007/s12011-024-04181-w)
Supplement: Supplementary file 1 — Supplementary Material 1 [file 12011_2024_4181_MOESM1_ESM.docx]

**LEEK**


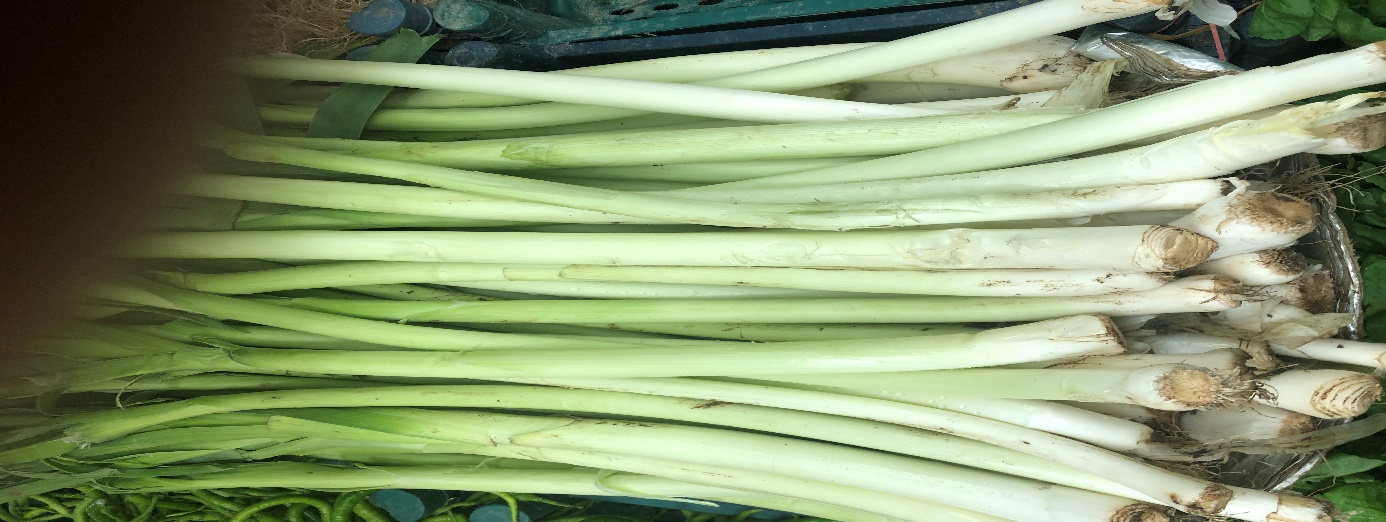


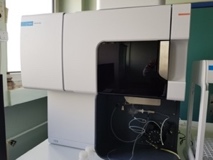


**Elements**

* P

* K

* C

*As

*Ba

*Pb

**Graphical Abstract:** In this study, the degree of accumulation of macro-, micro element and heavy metal contents of different parts and edible layers of leeks cultivated in Konya in Turkey was investigated. The elemental analysis results obtained with ICP-OES showed significant differences depending on the different parts of leek.
